# The epidemiology of pharmacologically treated attention deficit hyperactivity disorder (ADHD) in children, adolescents and adults in UK primary care

**DOI:** 10.1186/1471-2431-12-78

**Published:** 2012-06-19

**Authors:** Suzanne McCarthy, Lynda Wilton, Macey L Murray, Paul Hodgkins, Philip Asherson, Ian CK Wong

**Affiliations:** 1 School of Pharmacy, University College Cork, Cork, Ireland; 2 Pharmacy Department, Cork University Hospital, Cork, Ireland; 3 Centre for Paediatric Pharmacy Research, School of Pharmacy, University College London, London, UK; 4 Shire Pharmaceuticals LLC, Wayne, PA, USA; 5 MRC Social, Genetic and Developmental Psychiatry Centre, Institute of Psychiatry, London, UK; 6 Department of Pharmacology and Pharmacy, Li Ka Shing Faculty of Medicine, The University of Hong Kong, Hong Kong, Hong Kong

## Abstract

**Background:**

Attention Deficit Hyperactivity Disorder (ADHD) is a common neurodevelopmental disorder characterised by the symptoms of inattention, impulsivity and hyperactivity. ADHD was once perceived as a condition of childhood only; however increasing evidence has highlighted the existence of ADHD in older adolescents and adults. Estimates for the prevalence of ADHD in adults range from 2.5–4%. Few data exist on the prescribing trends of the stimulants methylphenidate and dexamfetamine, and the non-stimulant atomoxetine in the UK. The aim of this study was to investigate the annual prevalence and incidence of pharmacologically treated ADHD in children, adolescents and adults in UK primary care.

**Methods:**

The Health Improvement Network (THIN) database was used to identify all patients aged over 6 years with a diagnosis of ADHD/hyperkinetic disorder and a prescription for methylphenidate, dexamfetamine or atomoxetine from 2003–2008. Annual prevalence and incidence of pharmacologically treated ADHD were calculated by age category and sex.

**Results:**

The source population comprised 3,529,615 patients (48.9% male). A total of 118,929 prescriptions were recorded for the 4,530 patients in the pharmacologically treated ADHD cohort during the 6-year study. Prevalence (per 1000 persons in the mid-year THIN population) increased within each age category from 2003 to 2008 [6–12 years: from 4.8 (95% CI: 4.5–5.1) to 9.2 (95% CI: 8.8–9.6); 13–17 years: from 3.6 (95% CI: 3.3–3.9) to 7.4 (95% CI: 7.0–7.8); 18–24 years: from 0.3 (95% CI: 0.2–0.3) to 1.1 (95% CI: 1.0–1.3); 25–45 years: from 0.02 (95% CI: 0.01–0.03) to 0.08 (95% CI: 0.06–0.10); >45 years: from 0.01 (95% CI: 0.00–0.01) to 0.02 (95% CI: 0.01–0.03). Whilst male patients aged 6-12 years had the highest prevalence; the relative increase in prescribing was higher amongst female patients of the same age - the increase in prevalence in females aged 6–12 years was 2.1 fold compared to an increase of 1.9 fold for their male counterparts. Prevalence of treated ADHD decreased with increasing age. Incidence (per 1000 persons at risk in the mid-year THIN population) was highest for children aged 6–12 years.

**Conclusions:**

A trend of increasing prescribing prevalence of ADHD drug treatment was observed over the period 2003–2008. Prevalence of prescribing to adult patients increased; however the numbers treated are much lower than published estimates of the prevalence of ADHD. This study has added to the limited knowledge on ADHD prescribing in primary care, particularly in the area of drug treatment in adulthood.

## Background

Attention Deficit Hyperactivity Disorder (ADHD) is a common neurodevelopmental disorder characterised by the symptoms of inattention, impulsivity and hyperactivity. The prevalence of ADHD in school-aged children and adolescents in the United Kingdom (UK) using the broader Diagnostic and Statistical Manual of Mental Disorders, 4th Edition (DSM-IV) criteria is estimated at 5% [1]. The prevalence of hyperkinetic disorder (HKD) in children and young people (5–16 years), defined by the narrower International Classification of Disease 10th Revision (ICD-10) criteria is estimated at 1.5% [2].

ADHD/HKD affects children and adolescents in various ways and to different extents; however the consequences of the condition generally impact greatly on patients, their families and those around them. Untreated ADHD/HKD is frequently associated with underachievement in school, harm to relationships with family, teachers and friends, increased rates of criminality and accidents; and the development of comorbid psychiatric symptoms including oppositionality, anxiety, depression and substance misuse [3,4].

Treatments and interventions for ADHD/HKD are varied and include in the main, psychological therapies and pharmacological treatment [5]. When drug treatment is considered appropriate for the patient, the central nervous system stimulants methylphenidate (MPH) and dexamfetamine (DEX) and the non-stimulant atomoxetine (ATM) are recommended in the UK [1,5]. These drug treatments have been shown to improve the core symptoms of inattention, hyperactivity and impulsivity [5]. Methylphenidate, considered to be first-line therapy, has been used for over 50 years for the treatment of ADHD/HKD and is licensed in the UK for use as part of a comprehensive treatment programme for ADHD/HKD in children (over 6 years of age) and adolescents when remedial measures alone prove insufficient [6].

Only one previous study known to us has estimated incidence rates and the prevalence for pharmacologically treated ADHD in boys aged 5–14 years in the UK from 1996–2001 [7]. This study reported a prevalence of 5.3 per 1000 boys in 1999.

ADHD/HKD was once perceived as a condition of childhood only; however an increase in the evidence has highlighted the existence of ADHD/HKD in older adolescents and adults [5]. Estimates for the prevalence of the condition in adults range from 2.5–4% [4,8-10]. Whilst there is evidence of persistence of ADHD/HKD from childhood into adulthood [11], there are limited data on the treatment patterns of ADHD/HKD in adults in routine clinical practice. A cohort study in the UK examined the prevalence of prescribing of MPH, DEX and ATM in adolescents and young adults aged 15–21 years from 1999–2006 and identified a 6-fold increase in prescribing over this time. Prevalence of prescribing of these drugs to the older patients was significantly lower [12].

Currently, in the UK, Concerta® XL (prolonged-release MPH) is the only stimulant which has a license for use in the treatment of ADHD/HKD in adults, and only as a continuation treatment in patients whose symptoms have persisted from adolescence into adulthood and have shown clear benefit from treatment [6]. The non-stimulant ATM is also only indicated as continuation treatment in adults who started their treatment with this medication in childhood [13]. The published NICE guidelines (2008) [5] and the British Association for Psychopharmacology guidelines [14] strongly advocate that appropriate treatments, psychological or pharmacological, should be provided for adults with ADHD/HKD.

While it is recognised that the use of medications to treat childhood ADHD/HKD has increased in the last decade or so, which may be coincident with the publication of the NICE Technology Appraisal on methylphenidate in 2000 [15], there are limited data to support this and there are some concerns that ADHD/HKD treatments might be used inappropriately or over-prescribed [16]. To our knowledge only two studies have looked at pharmacologically treated ADHD in UK primary care [7,12]. These studies are limited as they report data from over a decade ago [7] and in restricted age groups; younger children [7] and older adolescents and young adults [12]. Therefore, this study aims to address the gaps in the literature and to estimate the prevalence and incidence of pharmacologically treated ADHD (MPH, DEX and ATM) in children (over 6 years), adolescents and adults in UK primary care.

## Methods

### Design and source population

A retrospective cohort study was performed using data from The Health Improvement Network (THIN). THIN contains anonymised computerised information entered by general practitioners (GPs) in the UK. With coverage of approximately 5.7% of the UK population (2009), practices that use the database are broadly representative of practices in the UK for patients’ characteristics [17]. GPs participating in THIN are trained to record information using the Vision general practice system (In Practice Systems; London, UK) and the validity of data on the database for research has been supported by a number of studies [18-20]. Data recorded in THIN include patient demographics, details from GP visits, diagnoses from specialist referrals and hospital admissions, and the results of laboratory tests. Prescriptions issued by the GP are directly generated from the computer. The Read classification is used to code specific diagnoses and related signs and symptoms, and a drug dictionary based on data from the MULTILEX classification is used to code drugs. Prescriptions issued by specialists are not coded onto the database but information on them may be available as free-text comments.

We identified all individuals who were aged 6 years and over and were registered with a GP practice on the THIN database between January 1, 2003 and December 31, 2008 (the study period). Age of individuals on the THIN database is calculated from the month and year of birth up to the age of 15 years. Once individuals reach the age of 15 years, age is calculated using the year of birth only (i.e. 1st January of that year).

The ‘start date’ for these individuals was calculated from the latest of three dates: the date they registered with the GP practice, the date the GP practice started to use the Vision practice system or the date of the practice’s Acceptable Mortality Reporting, used as a quality indicator for the practice. This information was obtained from patients’ records on THIN. This ‘start date’ could precede the start of the study period or could occur during the study period. Individuals were only included in the source population if they had an observation period of at least 12 months from their ‘start date’ and were registered during the study period.

### Identification of the pharmacologically treated ADHD cohort

The pharmacologically treated ADHD cohort comprised patients with both a prescription coded for a study drug during the study period and a diagnosis of ADHD coded at any time on the database (identified from Read codes). As THIN is a patient-records database, there is no requirement to have diagnoses coded every year. Therefore no time restriction was placed on when the diagnosis was recorded in relation to when the study drugs were prescribed. These patients were identified by examining the records of all individuals in the source population after each patient’s ‘start date’. The final pharmacologically treated ADHD cohort comprised 4,530 individuals.

### Prevalence calculation

The annual prevalence of pharmacologically treated ADHD was calculated by summing all patients with ADHD and a prescription for MPH, DEX or ATM (the study drugs) in a particular study year. This number was divided by the total number of individuals in the mid-year (1st July) source population in that calendar year. The annual prevalence was expressed per 1000 patients. Age-specific and sex-specific prevalences were calculated.

### Incidence calculation

The first 12 months following the patient’s ‘start date’ was used as a screening period. If the first prescription for MPH, DEX or ATM was identified during this 12-month screening period, it was not treated as the incident prescription. Patients prescribed ADHD drugs during this 12-month screening period were not included in the risk pool. Incident prescriptions were defined as a first prescription identified after this 12-month ‘screening period’ and hence the patient was identified as an incident patient during the year in which the first prescription was identified. Only those patients defined as incident patients during the study period were included in the numerator of this study.

### Incidence denominator

The denominator comprised individuals from the source population who were considered ‘at risk’ i.e. did not have a diagnosis of ADHD and a prescription for a study drug. Therefore, individuals’ data contributed to the denominator only after the first 12-month screening period following their ‘start date’. Patients who were prescribed ADHD medication before the study period were excluded from the denominator during the study period. Likewise, patients who were prescribed ADHD medications during the study period were excluded from the incidence denominator of subsequent years.

Annual incidence was calculated by dividing the number of incident patients by the total number of persons in the source population ‘at risk’ in the mid-year (1st July) population.

### Data analysis

Prevalence and incidence were calculated for each year of the study period 2003–2008 and stratified into different age bands (6–12 years, 13–17 years, 18–24 years, 25–45 years, >45 years). No formal comparative statistical analyses were performed.

Data manipulation and analysis were conducted using Stata/MP version 11.0 (StataCorp, College Station, Texas, United States).

### Ethical approval

Ethical approval for this study was granted by the Cambridgeshire 4 Research Ethics Committee (ref: 09/H0305/81).

## Results

The source population comprised 3,529,615 patients (48.9% male). A total of 118,929 prescriptions were recorded for the 4,530 patients aged ≥ 6 years in the pharmacologically treated ADHD cohort during the 6-year study period with a median number of prescriptions per patient of 17 [95% CI: 16, 18]. The average length of time that the 4,530 patients were registered on the database was 8.5 years [SD 4.7 years, range 1.0-21.1 years]. MPH was the drug most frequently prescribed during the study period followed by ATM and DEX (Table 1).

**Table 1:** Total number of prescriptions of methylphenidate, dexamfetamine and atomoxetine recorded from 2003 to 2008

	**2003**	**2004**	**2005**	**2006**	**2007**	**2008**	**Total**
Total Number of Prescriptions	11,441	14,763	17,906	22,108	26,205	26,506	118,929
Methylphenidate (n, % of total prescriptions)	11,053 (96.6)	14,233 (96.4)	16,058 (89.7)	19,710 (89.2)	23,255 (88.7)	23,476 (88.6)	107,785 (90.6)
Dexamfetamine (n, % of total prescriptions)	388 (3.4)	352 (2.4)	433 (2.4)	487 (2.2)	494 (1.9)	484 (1.8)	2,638 (2.2)
Atomoxetine (n, % of total prescriptions)	N/A*	178 (1.2)	1,415 (7.9)	1,911 (8.6)	2,456 (9.4)	2,546 (9.6)	8506 (7.2)

*N/A: no prescriptions for atomoxetine were issued on the database to a patient in this study in the year 2003; it was marketed in the UK starting 2004.

### Prevalence of treated ADHD

**Figure 1:**
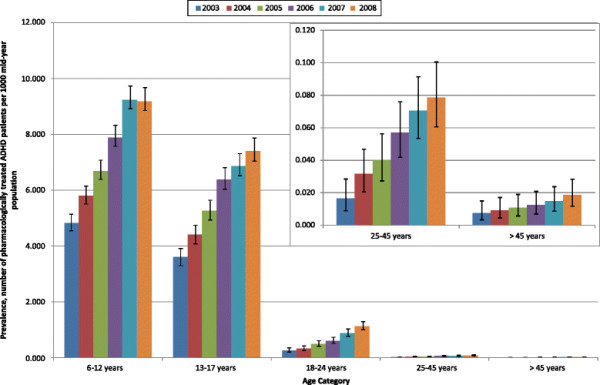
Prevalence of pharmacologically treated attention deficit hyperactivity disorder (methylphenidate, dexamfetamine or atomoxetine) in patients aged 6-years and over in UK general practice (with 95% confidence intervals).

There was a trend for the annual prevalence estimates to increase year on year from 2003 to 2008 (Table 2). Figure 1 demonstrates this increase in prevalence estimates over the study period and also that the prevalence estimates decreased with increasing age. The prevalence estimates approximately doubled between 2003 and 2008, in children and adolescents and those patients over 45 years old; however the largest increase was observed in adults aged 18–24 years and 25–45 years, with an approximate 4-fold increase in prevalence estimates.

**Table 2:** Annual prevalence (2003–2008) of pharmacologically treated ADHD (methylphenidate, dexamfetamine and atomoxetine) by age category and gender

**Age Category**	**Year**	**2003**	**2004**	**2005**	**2006**	**2007**	**2008**
Total Prevalence*1000 (Males and Females ≥ 6 years)		**0.709**	**0.862**	**1.014**	**1.203**	**1.365**	**1.403**
95% Cl (lb)*1000		0.676	0.825	0.975	1.161	1.320	1.358
95% Cl (ub)*1000		0.743	0.899	1.054	1.246	1.411	1.449
**6-12 years**							
Female Prevalence*1000		**1.339**	**1.642**	**2.042**	**2.310**	**2.775**	**2.769**
95% Cl (lb)*1000		1.131	1.411	1.784	2.034	2.470	2.464
95% Cl (ub)*1000		1.574	1.900	2.327	2.613	3.108	3.101
Male Prevalence*1000		**8.147**	**9.758**	**11.133**	**13.224**	**15.432**	**15.321**
95% Cl (lb)*1000		7.634	9.195	10.532	12.566	14.716	14.607
95% Cl (ub)*1000		8.685	10.346	11.759	13.907	16.174	16.061
Total Prevalence*1000		**4.825**	**5.795**	**6.688**	**7.886**	**9.241**	**9.181**
95% Cl (lb)*1000		4.543	5.485	6.355	7.522	8.844	8.786
95% Cl (ub)*1000		5.121	6.119	7.034	8.262	9.651	9.590
**13-17 years**							
Female Prevalence*1000		**0.635**	**0.947**	**1.106**	**1.446**	**1.767**	**2.002**
95% Cl (lb)*1000		0.465	0.742	0.890	1.201	1.497	1.714
95% Cl (ub)*1000		0.847	1.191	1.360	1.727	2.071	2.325
Male Prevalence*1000		**6.250**	**7.535**	**9.104**	**11.027**	**11.752**	**12.569**
95% Cl (lb)*1000		5.719	6.963	8.485	10.350	11.054	11.845
95% Cl (ub)*1000		6.818	8.141	9.757	11.737	12.483	13.325
Total Prevalence*1000		**3.608**	**4.404**	**5.265**	**6.378**	**6.865**	**7.396**
95% Cl (lb)*1000		3.314	4.087	4.925	6.008	6.484	6.999
95% Cl (ub)*1000		3.921	4.739	5.622	6.765	7.264	7.810
**18-24 years**							
Female Prevalence*1000		**0.118**	**0.116**	**0.112**	**0.244**	**0.337**	**0.335**
95% Cl (lb)*1000		0.059	0.058	0.056	0.158	0.236	0.237
95% Cl (ub)*1000		0.211	0.207	0.200	0.361	0.466	0.460
Male Prevalence*1000		**0.396**	**0.521**	**0.845**	**0.943**	**1.385**	**1.876**
95% Cl (lb)*1000		0.283	0.391	0.679	0.770	1.178	1.637
95% Cl (ub)*1000		0.540	0.680	1.038	1.142	1.619	2.140
Total Prevalence*1000		**0.263**	**0.327**	**0.493**	**0.607**	**0.878**	**1.122**
95% Cl (lb)*1000		0.196	0.253	0.402	0.507	0.759	0.989
95% Cl (ub)*1000		0.346	0.417	0.599	0.721	1.010	1.267
**25-45 years**							
Female Prevalence*1000		**0.008**	**0.026**	**0.028**	**0.040**	**0.045**	**0.052**
95% Cl (lb)*1000		0.002	0.012	0.014	0.023	0.027	0.032
95% Cl (ub)*1000		0.023	0.047	0.050	0.065	0.071	0.079
Male Prevalence*1000		**0.025**	**0.038**	**0.052**	**0.074**	**0.095**	**0.105**
95% Cl (lb)*1000		0.012	0.021	0.032	0.050	0.068	0.076
95% Cl (ub)*1000		0.046	0.062	0.079	0.105	0.130	0.141
Total Prevalence*1000		**0.017**	**0.032**	**0.040**	**0.057**	**0.070**	**0.079**
95% Cl (lb)*1000		0.009	0.021	0.027	0.042	0.053	0.061
95% Cl (ub)*1000		0.028	0.047	0.056	0.076	0.091	0.100
**>45 years**							
Female Prevalence*1000		**0.007**	**0.011**	**0.007**	**0.010**	**0.010**	**0.011**
95% Cl (lb)*1000		0.002	0.004	0.002	0.004	0.004	0.005
95% Cl (ub)*1000		0.018	0.023	0.018	0.022	0.022	0.024
Male Prevalence*1000		**0.008**	**0.008**	**0.015**	**0.015**	**0.020**	**0.027**
95% Cl (lb)*1000		0.002	0.002	0.007	0.006	0.010	0.015
95% Cl (ub)*1000		0.020	0.020	0.030	0.029	0.036	0.044
Total Prevalence*1000		**0.008**	**0.009**	**0.011**	**0.012**	**0.015**	**0.019**
95% Cl (lb)*1000		0.003	0.004	0.006	0.007	0.009	0.012
95% Cl (ub)*1000		0.015	0.017	0.019	0.021	0.024	0.028

CI (Confidence Interval), lb (lower boundary) and ub (upper boundary).

These patterns were also evident when the data were further stratified by gender (Table 2). For both genders and across all age categories (with the exception of patients aged over 45 years), the annual prevalence estimates increased from 2003–2008, with the prevalence being lower in female patients for all age categories. It is observed from Table 2 that the prevalence of prescribing to males and females over 45 years was very low. The number of patients aged 45 years and over in the patient dataset ranged from 8 in 2003 to 22 in 2008.

For male and female children aged 6–12 years, the prevalence increased 1.88 fold and 2.07 fold respectively. Prevalence estimates increased 2.01 fold for adolescent males aged 13–17 years, whereas prevalence estimates increased 3.15 fold for their female counterparts. For male and female patients aged 25–45 years the prevalence estimates increased 4.2 and 6.5 fold respectively during the study period. Table 3 presents the ratio of male to female prescribing prevalence and the ratio of males to females registered on the database across each of the age categories. Whilst the proportions of males and females on the database in the various age categories and over time were similar, a decrease in the ratio of prescribing prevalence estimates was observed for children aged 6–12 years, adolescents aged 13–17 years and adults aged 25–45 years. The data suggest that for these age categories, the rate of increase in prevalence was greater in females than males. Conversely, for young adults aged 18–24 years and for adults aged over 45 years, the ratio of prescribing increased thereby indicating that the rate of increase in prescribing to males was greater than that observed in females.

**Table 3:** Ratio of male to female prescribing prevalence by age category and study year and ratio of males to females registered on the database

**Age Category**	**2003**	**2004**	**2005**	**2006**	**2007**	**2008**
**Prevalence 6–12 years**	6.085	5.942	5.451	5.725	5.560	5.534
**Registered on database 6–2 years**	1.050	1.048	1.045	1.045	1.044	1.044
**Prevalence 13–17 years**	9.838	7.955	8.228	7.625	6.652	6.277
**Registered on database 13–17 years**	1.125	1.104	1.083	1.061	1.043	1.043
**Prevalence 18–24 years**	3.355	4.492	7.549	3.856	4.117	5.602
**Registered on database 18–24 years**	1.084	1.093	1.084	1.079	1.066	1.043
**Prevalence 25–45 years**	3.252	1.461	1.867	1.835	2.120	2.020
**Registered on database 25–45 years**	1.025	1.026	1.023	1.022	1.022	1.014
**Prevalence >45 years**	1.108	0.736	2.202	1.462	2.004	2.339
**Registered on database >45 years**	0.903	0.906	0.908	0.912	0.915	0.916

### Incidence of treated ADHD

**Figure 2:**
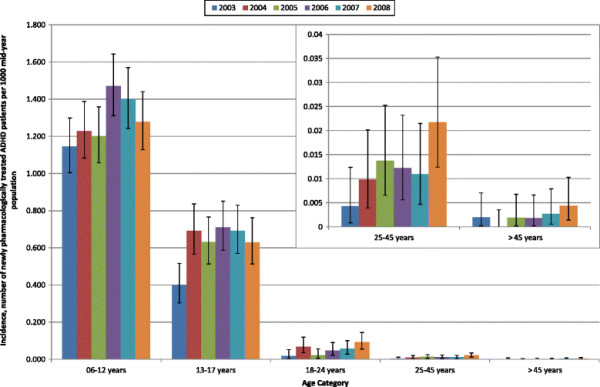
Incidence of pharmacologically treated attention deficit hyperactivity disorder (methylphenidate, dexamfetamine or atomoxetine) in patients aged 6-years and over in UK general practice (with 95% confidence intervals).

The source population for incidence calculations was 3,226,266 (49.3% male). A total of 2,343 patients were incident during the study period. Incidence of prescribing is illustrated in Figure 2. The analysis shows that the incidence estimates were highest for children (6–12 years) and decreased with increasing age, being very low in adults. The incidence estimates were higher in 2008 than in 2003 for all age groups, although the incidence estimates for children and for adolescents (13–17 years) were highest in 2006. The incidence estimates were much higher for young adults (18–24 years) in 2004, the year in which atomoxetine entered the market, than in all other years except for 2008. When the data were stratified by gender, the incidence estimates were shown to be much lower in females than in males (Table 4). Similarly to prevalence estimates, the incidence estimates increased proportionally more for female children and adolescents than for the males of these age categories. However for young adult males (aged 18–24 years) the increase was 7.23 fold compared to 1.62 fold for young adult female patients.

**Table 4:** Annual incidence (2003–2008) of pharmacologically treated ADHD (methylphenidate, dexamfetamine and atomoxetine) by age category and gender

**Age Category**	**Year**	**2003**	**2004**	**2005**	**2006**	**2007**	**2008**
**6-12 years**							
Female Incidence*1000		**0.272**	**0.388**	**0.358**	**0.425**	**0.433**	**0.424**
95% Cl (lb)*1000		0.181	0.277	0.252	0.309	0.314	0.307
95% Cl (ub)*1000		0.393	0.528	0.494	0.571	0.581	0.571
Male Incidence*1000		**1.982**	**2.036**	**2.013**	**2.476**	**2.331**	**2.101**
95% Cl (lb)*1000		1.725	1.776	1.753	3.188	2.049	1.833
95% Cl (ub)*1000		2.267	2.325	2.300	2.793	2.642	2.397
Total Incidence*1000		**1.145**	**1.229**	**1.201**	**1.470**	**1.400**	**1.278**
95% Cl (lb)*1000		1.005	1.084	1.058	1.311	1.243	1.128
95% Cl (ub)*1000		1.300	1.388	1.359	1.643	1.570	1.441
**13-17 years**							
Female Incidence*1000		**0.102**	**0.178**	**0.168**	**0.224**	**0.318**	**0.270**
95% Cl (lb)*1000		0.0471	0.095	0.090	0.133	0.208	0.169
95% Cl (ub)*1000		0.210	0.304	0.288	0.354	0.465	0.409
Male Incidence*1000		**0.666**	**1.160**	**1.063**	**1.174**	**1.052**	**0.977**
95% Cl (lb)*1000		0.496	0.937	0.852	0.954	0.845	0.777
95% Cl (ub)*1000		0.876	1.422	1.309	1.429	1.295	1.212
Total Incidence*1000		**0.400**	**0.691**	**0.631**	**0.710**	**0.691**	**0.629**
95% Cl (lb)*1000		0.303	0.566	0.514	0.587	0.571	0.514
95% Cl (ub)*1000		0.516	0.836	0.767	0.851	0.829	0.762
**18-24 years**							
Female Incidence*1000		**0.013**	**0.037**	**0.000**	**0.023**	**0.022**	**0.021**
95% Cl (lb)*1000		0.000	0.008	0.000	0.003	0.003	0.002
95% Cl (ub)*1000		0.071	0.108	0.044	0.082	0.079	0.074
Male Incidence*1000		**0.022**	**0.096**	**0.041**	**0.070**	**0.087**	**0.159**
95% Cl (lb)*1000		0.003	0.044	0.011	0.028	0.040	0.092
95% Cl (ub)*1000		0.080	0.182	0.106	0.144	0.165	0.254
Total Incidence*1000		**0.018**	**0.069**	**0.022**	**0.048**	**0.056**	**0.093**
95% Cl (lb)*1000		0.004	0.036	0.006	0.022	0.028	0.056
95% Cl (ub)*1000		0.052	0.120	0.057	0.091	0.101	0.145
**25-45 years**							
Female Incidence*1000		**0.000**	**0.009**	**0.006**	**0.011**	**0.003**	**0.016**
95% Cl (lb)*1000		0.000	0.002	0.001	0.003	0.000	0.006
95% Cl (ub)*1000		0.011	0.025	0.020	0.028	0.015	0.036
Male Incidence*1000		**0.008**	**0.011**	**0.022**	**0.013**	**0.019**	**0.027**
95% Cl (lb)*1000		0.002	0.003	0.009	0.004	0.008	0.013
95% Cl (ub)*1000		0.024	0.028	0.043	0.031	0.039	0.049
Total Incidence*1000		**0.004**	**0.010**	**0.014**	**0.013**	**0.011**	**0.022**
95% Cl (lb)*1000		0.001	0.004	0.007	0.006	0.005	0.012
95% Cl (ub)*1000		0.012	0.020	0.025	0.023	0.021	0.035
**>45 years**							
Female Incidence*1000		**0.004**	**0.000**	**0.000**	**0.002**	**0.002**	**0.000**
95% Cl (lb)*1000		0.000	0.000	0.000	0.000	0.000	0.000
95% Cl (ub)*1000		0.013	0.007	0.007	0.10	0.010	0.006
Male Incidence*1000		**0.000**	**0.000**	**0.004**	**0.002**	**0.004**	**0.009**
95% Cl (lb)*1000		0.000	0.000	0.000	0.000	0.000	0.003
95% Cl (ub)*1000		0.008	0.007	0.014	0.011	0.014	0.021
Total Incidence*1000		**0.002**	**0.000**	**0.002**	**0.002**	**0.003**	**0.004**
95% Cl (lb)*1000		0.000	0.000	0.000	0.000	0.001	0.001
95% Cl (ub)*1000		0.007	0.004	0.007	0.007	0.008	0.010

## Discussion

In this study, estimates for the prevalence of MPH, DEX and ATM prescribing were calculated for the period, 2003 to 2008, using data from THIN database. It was observed that prevalence of prescribing increased over this time, with a ~2-fold increase for children and adolescents, and a 4–5-fold increase for adults. Incidence of prescribing showed similar patterns whereby the incidence of prescribing was greater in children and declined in adulthood.

### School-age children and adolescents

Overall, prevalence increased over the study period from 4.83 to 9.18 per 1000 patients aged 6–12 years. The highest prevalence of prescribing in this study was to boys aged 6–12 years (15.32 per 1000 boys aged 6–12 years in 2008). Prescribing to male patients in this age category was higher than to female patients (6:1 in 2003; 5.5:1 in 2008). These findings are in line with figures reported in the literature, which report differences in prescribing between the genders ranging from a ratio of 2:1 to 9 [21]. It is not known to what extent this is a true behavioural gender difference and how much is due to factors such as the under-diagnosis and under-reporting of the condition in females. Interestingly, the relative increase in prevalence over the study for children 6–12 years was slightly higher in female patients compared to male patients (2.1 compared with 1.9 times increase). A similar observation was reported by Cox et al., who reported that in the US from 2002 to 2005, the rate of growth of ADHD drug prescribing to females was double that of males [22].

Treatment prevalence for adolescents aged 13–17 years followed a similar pattern whereby the overall prevalence doubled over the study period. The driver of this increase was prescribing to adolescent girls (3.15 increase over the 6 year period) although actual prevalence was again higher in male patients.

To our knowledge, only one previous study has examined the use of these drugs in children in the UK [7]. The authors reported on the incidence and prevalence of MPH and DEX in boys aged 5–14 years from 1996–2001. This study reported a prevalence of 5.3 per 1000 boys in 1999.

A study from the Netherlands used computerised pharmacy dispensing records to examine the prevalence and incidence of psychotropic medications in Dutch children from 1995 to 1999. The highest prevalence was seen in children aged 5–9 years which, in 1999, was 13.9 per 1000 children [23]. More recently, a retrospective analysis was conducted by Hodgkins and colleagues to estimate the incidence and prevalence of children, aged 6–17 years, receiving initial pharmacotherapy for ADHD between 2000 and 2007 from a large sample representative of the general population of the Netherlands [24]. Data extrapolated from the PHARMO database to the Netherlands population demonstrated an increase in yearly incidence from 30 per 10,000 in the year 2000 to 75 per 10,000 in the year 2007. Prevalence increased from 110 per 10,000 in 2000 to 210 per 10,000 in 2007 [24].

A study examining prescribing trends for stimulants from 1992 to 1998 using North Carolina Medicaid prescription claim files reported an increase in prevalence from 44 per 1000 patients in 1992 to 95 per 1000 patients in 1998 in children aged 6–14 years. The authors of this paper acknowledged that the rates observed in their study were much higher than other studies reported; however they do not speculate as to why this is the case [25].

More recent studies from the US include a study by Zuvekas et al. who used the Medical Expenditure Panel Survey database to report prevalence of stimulant use from 1997 to 2002 in children aged less than 19 years [26]. The prevalence increased from 27 per 1000 patients (95% CI: 23–31 per 1000) in 1997 to 29 per 1000 patients (95% CI: 25–33 per 1000) in 2002. They also reported the highest use of stimulants in children aged 6–12 years. Cox et al. used ambulatory prescription claims data of children aged 5–19 years from 2002 to 2005 and over this period reported a growth in prevalence of ADHD medications of 40.4% [22].

These utilisation studies suggest that especially in the US, the prevalence of stimulant use increased substantially during the last decade.

The current study has demonstrated a trend of increasing prevalence of pharmacological treatments in the UK, throughout the study period; however the highest prevalence figure reported of 15.4 per 1000 male patients aged 6–12 years is in line with or below those reported in both the Netherlands and the US.

More importantly this figure is also lower than the global prevalence of ADHD in children or that of hyperkinetic disorders in the UK, which were recently estimated to be 5% and 1.5% respectively [2,27]. This is relevant as current NICE clinical guidelines recommend that for school-age children and young people with severe ADHD (hyperkinetic disorder), drug treatment should be offered as the first-line treatment and that medication will also be appropriate for patients with moderate levels of impairment who have refused non-drug interventions, or whose symptoms have not responded sufficiently to parent-training/education programmes or group psychological treatment [5].

### Adults

Population surveys in adult populations estimate the prevalence of ADHD in adults to be between 2.5 and 4% [4,8-10]. Whilst not all patients will require pharmacological intervention, NICE recommends that it should be the first-line treatment unless the person would prefer a psychological approach [5]. The results of this study suggest a trend of increasing prevalence of prescribing of ADHD drugs to adult patients; however the numbers remain much lower than the estimated prevalence of the condition. There may be several reasons for this including that earlier NICE guidelines in 2000 [15] indicated that medication should be tailed off in adolescence and the lack of licensed medicines for the treatment of ADHD in adulthood. It is expected that with the recommendation in the current NICE guidelines [5] for pharmacological treatment of adult ADHD, the increase in prevalence seen in this study will increase further and this may better reflect the prevalence of the disorder in a few years' time.

### Strengths and limitations

A significant strength of the study was the use of a large database such as THIN which provided primary care data on a cohort of over 4500 patients. THIN has been used widely in epidemiological research, including studies on mental health [28,29]. The use of THIN data allowed us to capture what is actually happening under normal conditions of clinical practice, rather than in selected samples of patients recruited into clinical trials. NICE guidelines recommend that although medications should be initiated by healthcare professionals with appropriate expertise in ADHD, GPs may continue prescribing and monitoring of medications; thus the use of a general practice database is a suitable data source for identifying and examining ADHD prescribing patterns. Nevertheless, these data might underestimate the overall prescribing rates in the UK since in some regions of the country specialist mental health teams or paediatricians remain the main prescribers and some GP practices will not prescribe medications for ADHD. This might particularly influence the estimates for prescribing to adults, since prescribing practice for those over the age of 18 years is still not well established in the UK and prescribing by specialists rather than GPs is still the norm in many regions.

A limitation of the data is that detailed information on the diagnoses was not readily accessible; therefore it was not possible to determine the severity of ADHD in the patients identified. An inclusion criterion for the study was that patients were required to be registered on the database during the study period 2003–2008 and have a minimum of one year of registration on the database. However, patients may have registered on the database at various points during this period or before this period. Therefore, the amount of follow-up time for patients registered later on the database may have been less than those registered earlier in the study period. For incidence calculations, patients prescribed ADHD drugs during this 12-month screening period were not included in the risk pool for subsequent years of the study. This resulted in varying look-back periods, in that in 2003, patients incident in 2002 were removed, whereas in 2008, six years of incident patients were removed. However, as the numbers of incident patients are small relative to the denominator, it is unlikely that this would influence reported rates significantly. A potential bias in the data when comparing prevalence between 2003 and 2008 is that some patients may contribute data in both years. Data from such patients would not contribute to the change in prevalence. This potential overlap in the cohorts was not taken into account when comparing the prevalence between 2003 and 2008.

The issue of over-prescribing of ADHD medicines was outside the remit of this study, as studies which have looked at this question have identified patients with an ADHD diagnosis who do and do not receive medication and/or psychological treatments, along with patients who receive medication but who do not meet the diagnostic criteria for ADHD [30,31]. The detailed specialist records required to examine this are not routinely available for all patients on a general practice database such as THIN and so the current study cannot answer the question as to whether the stimulants (MPH, DEX and ATM) are over-prescribed. However, despite the increase in prescribing observed over the study period, the difference between the prevalence of the condition reported in the literature and the prevalence in this study of prescribing of these drugs to those with ADHD does provide some assurance that it is unlikely that these drugs are over-used in the UK. ADHD pharmacological treatments are generally accepted to have a favourable risk/benefit ratio but long-term safety of these drugs should continue to be monitored [32,33]. This has been identified as a priority for research for the European Union who have commissioned the ADDUCE study (http://adhd-adduce.org), as part of the Seventh Framework Programme for Research and Technological Development, to examine the long-term safety of stimulants.

## Conclusions

Epidemiological data from the THIN database revealed a trend of increasing prescribing prevalence of ADHD drug treatment over the period 2003–2008 overall and for all age groups. Whilst male patients aged 6–12 years had the highest prevalence, the relative increase in prescribing was higher amongst female patients of the same age. Prevalence of prescribing of ADHD drugs to adult patients increased over the six-year period; however the numbers remain much lower than the published estimates of the prevalence of the condition. Of the three study drugs, MPH had the highest prevalence, followed by ATM. DEX use in children declined over the study period. Incidence of ADHD drug prescribing was very low across all age categories.

This study has added to the existing knowledge on ADHD prescribing patterns in primary care; in particular to the area of ADHD drug treatment in adulthood.

## Competing interests

LW and MM declare that they have no competing interests. SM received research funding as a result of involvement in this research study. PH is an employee and stock holder of Shire Pharmaceuticals. Shire Pharmaceuticals develops and manufactures drugs to treat ADHD. PA has acted in an advisory role for Shire, Janssen-Cilag, Eli Lilly and Flynn Pharma. He has received education or research grants from Shire, Janssen-Cilag and Eli-Lilly. He has given talks at educational events sponsored by the above companies. ICKW was a member of National Institute for Health and Clinical Excellence (NICE) ADHD Guideline Group and British Association for Psychopharmacology ADHD guideline group. ICKW has received research grants from various pharmaceutical companies; including Shire Pharmaceuticals. He has given talks at educational events sponsored by Janssen-Cilag and Eli-Lilly and acted as an advisor to Shire.

## Authors' contributions

SM carried out the data analysis, interpretation and drafted the manuscript. LW contributed to the data analysis and interpretation; assisted with drafting the manuscript; reviewed the final manuscript. MM contributed to the study design, initial data analysis and review of the manuscript. PH conceived the need for the study, participated in the study design, and development of the manuscript. PA contributed to initiating the project, conceiving the study and study questions and review and write up of the paper. IW contributed to initiating the study, conceiving the study and study questions, and also review and write-up of the manuscript. All authors read and approved the final manuscript.

## Pre-publication history

The pre-publication history for this paper can be accessed here:

http://www.biomedcentral.com/1471-2431/12/78/prepub
